# Zebrafish endochondral growth zones as they relate to human bone size, shape and disease

**DOI:** 10.3389/fendo.2022.1060187

**Published:** 2022-12-06

**Authors:** Pierre Le Pabic, Daniel B. Dranow, Diego J. Hoyle, Thomas F. Schilling

**Affiliations:** ^1^ Department of Biology and Marine Biology, University of North Carolina Wilmington, Willmington, NC, United States; ^2^ Department of Developmental and Cell Biology, University of California, Irvine, Irvine, CA, United States

**Keywords:** Danio rerio, endochondral, skeleton, cartilage, growth plate

## Abstract

Research on the genetic mechanisms underlying human skeletal development and disease have largely relied on studies in mice. However, recently the zebrafish has emerged as a popular model for skeletal research. Despite anatomical differences such as a lack of long bones in their limbs and no hematopoietic bone marrow, both the cell types in cartilage and bone as well as the genetic pathways that regulate their development are remarkably conserved between teleost fish and humans. Here we review recent studies that highlight this conservation, focusing specifically on the cartilaginous growth zones (GZs) of endochondral bones. GZs can be unidirectional such as the growth plates (GPs) of long bones in tetrapod limbs or bidirectional, such as in the synchondroses of the mammalian skull base. In addition to endochondral growth, GZs play key roles in cartilage maturation and replacement by bone. Recent studies in zebrafish suggest key roles for cartilage polarity in GZ function, surprisingly early establishment of signaling systems that regulate cartilage during embryonic development, and important roles for cartilage proliferation rather than hypertrophy in bone size. Despite anatomical differences, there are now many zebrafish models for human skeletal disorders including mutations in genes that cause defects in cartilage associated with endochondral GZs. These point to conserved developmental mechanisms, some of which operate both in cranial GZs and limb GPs, as well as others that act earlier or in parallel to known GP regulators. Experimental advantages of zebrafish for genetic screens, high resolution live imaging and drug screens, set the stage for many novel insights into causes and potential therapies for human endochondral bone diseases.

## 1 Introduction

Research on the growth plates (GPs) of endochondral bones in mice has greatly impacted our understanding of skeletal development as well as the causes of human skeletal disorders. Early studies showed that the epiphyses of limb long bones remain cartilaginous and proliferative, thereby allowing bone growth (1). Genetic studies showed mechanisms regulating cartilage maturation, gradual replacement by osteoblasts, matrix deposition and continuous bone remodeling by osteoclasts (2). These discoveries laid the groundwork for much of modern skeletal research. Given the limited knowledge of the cellular and molecular mechanisms regulating the huge variety of sizes and shapes of other bones, such as those of the skull or vertebrae, much of our current understanding of skeletal development is based on work on GPs of long bones in the tetrapod limb.

Over the past several decades, the zebrafish has become a powerful system for genetic analysis of skeletal development. Despite having fins that lack the long bones found in tetrapod limbs and many other obvious anatomical differences in their skeletons, zebrafish have the same array of skeletal cell types found in humans. Furthermore, the work that has been done to date has shown that the molecular mechanisms that control skeletal development, growth and physiology are largely conserved despite over 400 million years since their lineages diverged from a common ancestor (3).

In this review, we present an overview of skeletal research in zebrafish with a special focus on endochondral growth zones (GZs), defined as regions of cartilage proliferation and maturation, which include the well-known GPs of long bones. For reviews covering other aspects of skeletal research in zebrafish (e.g. osteoblasts/osteoclasts, intramembranous skull bones, fin rays, scales), we refer the reader to the following (4–11). First, we provide a brief introduction to adult zebrafish skeletal anatomy with a specific focus on similarities with human endochondral bones. Next, we present the cellular architecture of GZs between zebrafish and humans and across the three major skeletal regions, cranial, axial and appendicular. Third, we compare endochondral development and physiology between zebrafish and mammals and review key recent studies that have led to insights into conserved cellular pathways that control bone size and shape in health and disease.

## 2 Skeletal anatomy in adult zebrafish and humans

### 2.1 Anatomical distribution of endochondral and intramembranous bones

#### 2.1.1 Modes of ossification

**Figure 1:**
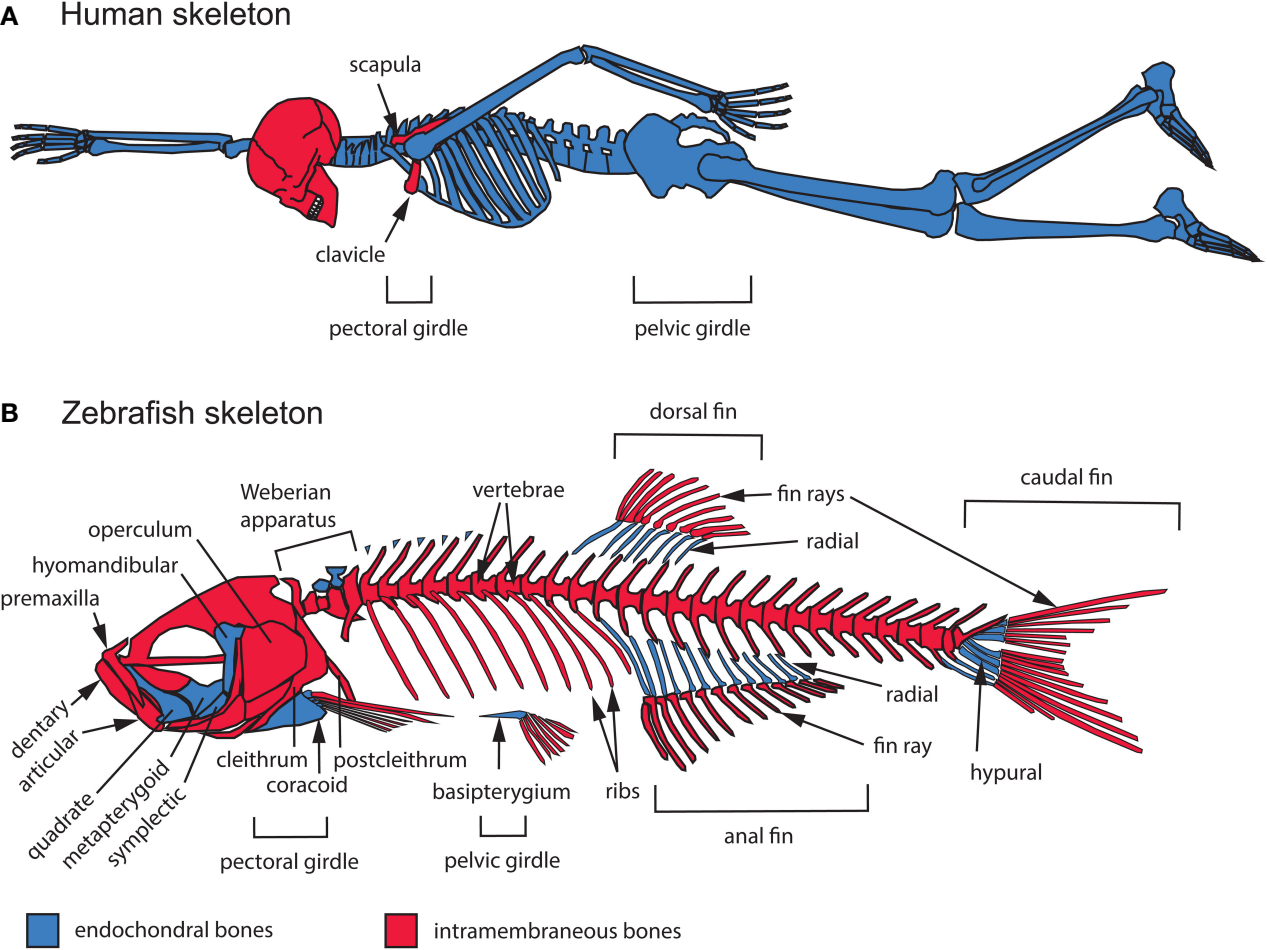
General overview of the intramembranous and endochondral composition of the zebrafish and human skeletons. **(A)** Human adult skeleton. In the head, intramembranous bones such as those of the calvaria (the top portion of the neurocranium) dominate the human skull in surface area, while endochondral bones mostly occupy the cranial base. All of the bones that compose the trunk and appendicular skeletons are endochondral, except for portions of the clavicle and scapula. **(B)** Zebrafish adult skeleton. The zebrafish skull, trunk and appendage skeletons are composed of both intramembranous and endochondral bones. The zebrafish skull is composed of 134 bones, 78 of which are endochondral. The zebrafish trunk skeleton is composed of intramembranous vertebrae and ribs. The zebrafish appendage skeleton is composed mostly of endochondral bones, while the fin ray exoskeleton is completely intramembranous.

Two modes of ossification produce the vertebrate skeleton: endochondral and intramembranous. In endochondral ossification, typified by long bones of the mammalian limb, mesenchymal condensations differentiate into cartilage that is eventually replaced by bone (2). In contrast, intramembranous bones, such as those of the skull vault, differentiate directly from mesenchyme (12). Some bones form by a combination of intramembranous and endochondral ossification, such as mammalian clavicles (13). The relative contributions of these two modes of ossification vary widely across different taxa, both in the axial skeleton, which consists of bones associated with the craniofacial complex and vertebrae, as well as the appendicular skeleton that supports the limbs and fins (**Figure 1**). Human and zebrafish skulls are both composed of a mixture of endochondral and intramembranous bones (14). While the mammalian calvaria occupies a large surface area, the chondrocranium and pharyngeal skeleton are composed of many smaller endochondral bones, just as in zebrafish (14, 15). In contrast, most of the zebrafish vertebral and limb skeletons are intramembranous while they are endochondral in humans. Despite these differences, zebrafish and humans are generally very similar in their development and basic structure. However, homologies between individual axial and appendicular bones of teleost fish and humans can be difficult to determine due to phylogenetic divergence and adaptation to different environments.

#### 2.1.2 Bones of the axial and appendicular skeletons

In the skull, difficulty in identifying homologous bones between humans and other vertebrates is thought to be partly a consequence of progressive fusion of skeletal elements during mammalian evolution (16). The human skull contains 29 bones, all joined by fibrous joints known as sutures, except for the mandible, hyoid bone, and middle ear ossicles (17). Two thirds of these cranial bones are intramembranous, while the hyoid bone, middle ear ossicles, and several bones of the cranial base (ethmoid, body and lesser wings of the sphenoid, petrous portion and otic capsule of the temporal bone, and basal portion of the occipital bone) are endochondral (**Figure 1**). In contrast, the zebrafish skull contains 134 bones, 78 of which are endochondral (14). As in humans, the intramembranous bones of the zebrafish braincase suture together, while bones supporting the jaws, opercle, gills and other parts of the skull, articulate with each other by mobile joints (**Figure 1**).

The non-cranial portion of the axial skeleton includes the vertebral column and rib cage in both humans and zebrafish, in addition to the unpaired fins (dorsal, anal, caudal) in zebrafish (**Figure 1**). Vertebrae and ribs are endochondral in humans but intramembranous in zebrafish (4, 18–20). In addition, unlike in humans, the zebrafish ribcage remains open ventrally and lacks a sternum. The zebrafish axial skeleton also includes appendages with no homologs in humans: the dorsal, anal, and caudal fins. Fins consist of an exoskeleton of rays made of intramembranous bone, and a supporting internal skeleton made of endochondral hypurals in the caudal fin and radials in the dorsal and anal fins (**Figure 1B**). Lastly, the Weberian apparatus, an evolutionary innovation linking the ear to the swim bladder to enhance audition (a character found only in the Ostariophysan superorder), contains both intramembranous and endochondral bones (21) (**Figure 1B**).

Human and zebrafish appendicular skeletons consist of pectoral (shoulder) and pelvic (hip) girdles with associated appendages: fore- and hindlimbs in humans, pectoral and pelvic fins in zebrafish. Human limbs are entirely composed of endochondral bones, while paired fins in zebrafish consist of fin rays made of intramembranous bone supported proximally by endochondral radial bones (22). In humans, most of the pectoral and pelvic girdles are also endochondral, though portions of the clavicle (collar bone) and scapula (shoulder blade) form by intramembranous ossification (**Figure 1A**). Similarly, the zebrafish pectoral girdle contains a mixture of intramembranous (cleithrum, postcleithrum, supracleithrum) and endochondral (coracoid, mesocoracoid, scapula) bones, while the pelvic girdle is exclusively endochondral (basipterygium) (14).

### 2.2 Endochondral growth zone structure

#### 2.2.1 Cellular architecture of endochondral growth zones

**Figure 2:**
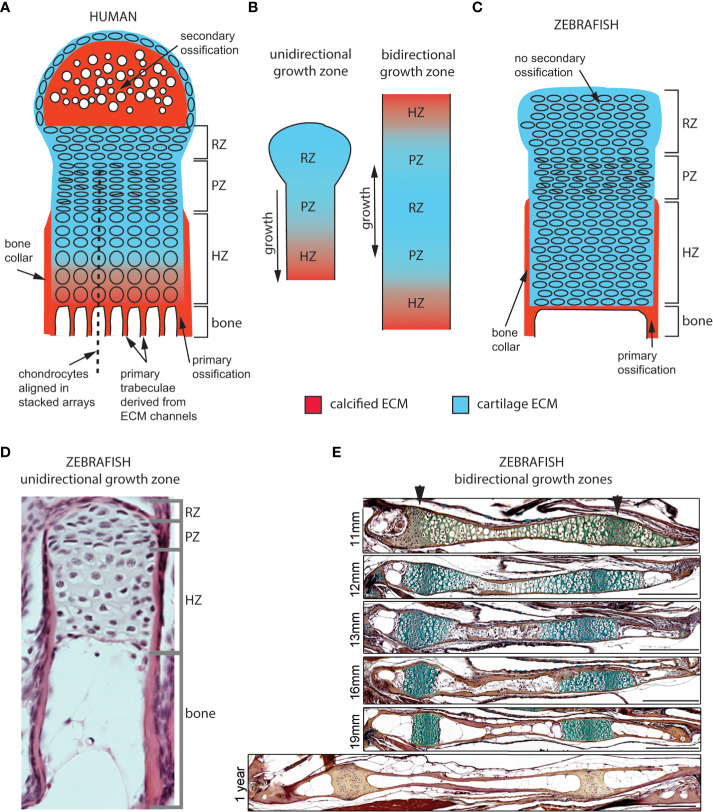
Cellular organization of epiphyseal and synchondroseal growth zones. **(A)** Human growth plate chondrocytes transition through resting-, proliferative- and hypertrophic zones (RZ, PZ, and HZ, respectively) before dying or transitioning to an osteoblast fate at the chondro-osseous junction. Cartilage cells stop dividing and enlarge in the hypertrophic zone. The bone collar forms a sheath around hypertrophic chondrocytes; the secondary ossification flanks the growth plate distally. Primary bone trabeculae derived from extracellular matrix channels populate the bone cavity. **(B)** In unidirectional (epiphyseal) growth zones, the resting zone is distal to the proliferative zone, itself distal to the hypertrophic zone; this layout produces unidirectional growth. In bidirectional (synchondroseal) growth zones, the resting zone is flanked by two proliferative zones and two hypertrophic zones in a mirror image organization; this layout produces bidirectional growth. **(C)** Stereotypical zebrafish unidirectional growth zone organization: chondrocytes transition through RZ, PZ and HZ, but they do not enlarge in the HZ. At the zebrafish resorption front, chondrocytes die or transition to either an osteocyte or adipocyte fate. A perichondral bone collar sheathes the zebrafish hypertrophic zone, but no secondary ossification is associated with zebrafish epiphyseal growth zones. Trabeculae are not observed in smaller teleosts such as zebrafish. **(D)** Histological section of zebrafish proximal radial showing unidirectional endochondral growth zone [originally published in (23)]. **(E)** Time series of maturation at two zebrafish bidirectional growth zones located within the ventral (left) and dorsal (right) ceratohyal synchondroses [originally published in (24)]. (scale bars = 50 µm).

In endochondral GZs, step-by-step chondrocyte maturation regulates bone elongation (**Figure 2**) (1). The maturation process starts in the resting zone (RZ), which serves the role of stem-cell niche (**Figure 2A**). Slow-dividing RZ cells transit into the proliferative zone (PZ), where they proliferate at a higher rate and stack to form chondrocyte arrays characteristic of avian and murine long bone GPs. They subsequently stop dividing and enlarge as they enter the hypertrophic zone (HZ). Most undergo apoptosis at the chondro-osseous junction and are subsequently replaced by bone. In GPs with steady-state growth, pools of cells in each zone remain constant as: 1) the rate of PZ cell division offsets the rate of cells leaving the PZ, 2) the rate of cells leaving offsets the rate of cells entering the PZ, and 3) the rate of cells entering the HZ offsets the rate of cells lost at the chondro-osseous junction (25). These aspects of cartilage maturation appear broadly similar between mammalian and zebrafish endochondral GZs, though chondrocytes are not aligned into linear stacks in zebrafish PZs (26, 27).

**Figure 3:**
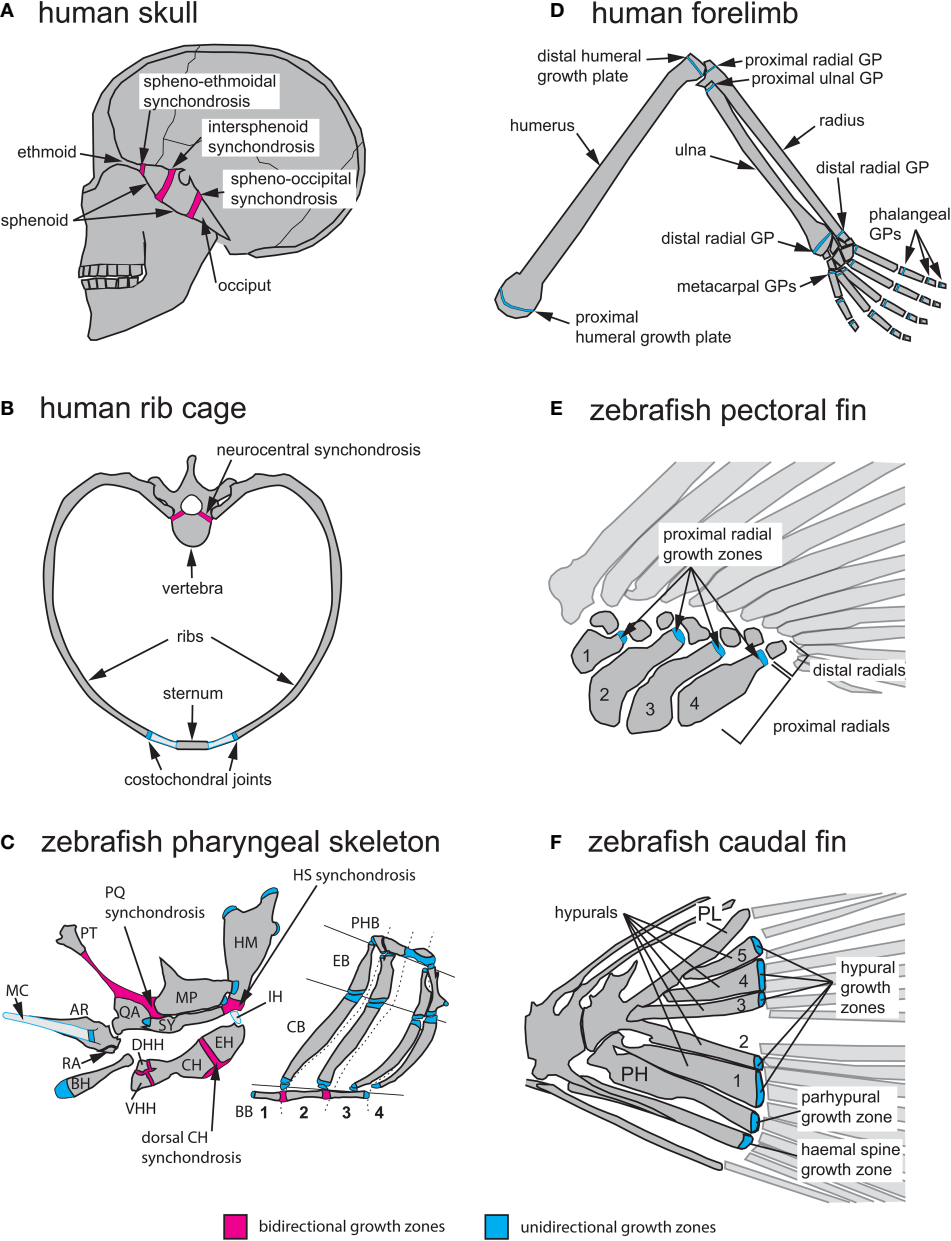
Anatomical locations of endochondral growth zones in human vs zebrafish. **(A)** Three synchondroses mediate growth of the human skull base (depicted in sagittal view): the spheno-ethmoidal-, intersphenoid- and spheno-occipital synchondroses. **(B)** Endochondral growth of human vertebrae and ribs (depicted in transverse view) takes place at neurocentral synchondroses and costochondral joints, respectively. **(C)** Over thirty endochondral growth zones mediate zebrafish pharyngeal skeleton growth. Three highly visible synchondroses mediate growth of first and second pharyngeal arch (PA1-2) skeletons. The PQ synchondrosis mediates growth of the QA and MP. The HS synchondrosis mediates growth of the SY and HM. The dorsal CH synchondrosis mediates growth of the CH and EH. In PA1 and 2, epiphyseal growth zones are found at the posterior MP, anterior SY, dorsal HM and anterior BH. In the gill supporting skeleton, epiphyseal growth zones are found at the ends of each CB and EB bone. **(D)** Axial growth of human stylopod (humerus, femur) and zeugopod (radius, ulna, tibia, fibula) bones is mediated by epiphyseal growth zones (=growth plates) located at each bone extremity. A single proximal epiphyseal growth zone mediates axial elongation of each autopod long bone (hand and foot phalanges). **(E)** In the zebrafish pectoral fin, epiphyseal growth zones are found at the distal end of each proximal radial. **(F)** In the zebrafish caudal fin, epiphyseal growth zones located at the distal end of each hypural bone (H1-5), the prehypural and two last haemal spines mediate axial elongation. AR, articular; BB, basibranchials; BH, basihyal; CB, ceratobranchial; CH, ceratohyal; DHH, dorsal hypohyal; EB, epibranchial; EH, epihyal; HM, hyomandibular; HS, hyosymplectic; IH, interhyal; MC, Meckel’s cartilage; MP, metapterygoid; PH, parhypural; PHB, pharyngobranchials; PL, pleurostyle; PT, palatine; QA, quadrate; RA, retroarticular; SY, symplectic; VHH, ventral hypohyal. Red indicates bidirectional- and blue indicates unidirectional growth zones.

Cartilage maturation can occur in one or both directions at GZs, parallel to the long axis of bone growth. In unidirectional (or epiphyseal) GZs (also known as GPs) typical of long bones, the RZ lies close to the distal-most region of the bone (epiphysis) and maturing cells progress medially toward the bone’s central shaft (diaphysis), producing axial elongation at each end (1). In contrast, bidirectional GZs produce growth in two opposite directions (28). This reflects a mirror-image organization where two sets of PZs and HZs flank a single RZ on either side (**Figure 2B**). Bidirectional GZs are often located within synchondroses or cartilaginous joints. In humans, they can be found in the skull base and vertebrae but in zebrafish are more common and found in multiple endochondral bones of the neurocranium and pharyngeal skeleton (**Figure 3**) (27).

#### 2.2.2 Tissue architecture of endochondral bones

Although both human and zebrafish endochondral bones have GZs, they show several structural differences, including the fact that zebrafish lack: 1) secondary ossifications, 2) trabecular bone, and 3) a hematopoietic bone marrow (**Figure 2**). Human GPs often have “secondary” ossification centers distal to the RZ, which appear later in endochondral differentiation (**Figure 2A**) (2). In contrast, in zebrafish and other teleost GZs, maturing cartilage remains continuous with articular cartilage at the joints, similar to earlier stages of mammalian GP development (**Figures 2C–E**) (27, 29, 30). Secondary ossification centers in mammals were recently proposed to have evolved to protect hypertrophic chondrocytes from mechanical damage in load-bearing tetrapod bones (31). Another striking structural difference from mammals is the absence of primary bone trabeculae at the resorption front in zebrafish (32, 33). Primary trabeculae form parallel bone channels in mammals through the progressive replacement of extracellular matrix (ECM) tracks produced by chondrocyte stacks by bone ECM (**Figure 2A**), while secondary trabeculae appear later in response to mechanical stress (34, 35). Thus, the less well-aligned chondrocyte stacks of zebrafish GZs as well as the lower amount of ECM produced by GZ chondrocytes (also observed in other teleosts) may help explain the lack of primary trabeculae (**Figures 2C–E**) (29, 30, 36). However, trabeculae have been reported in the bones of larger teleosts, suggesting that their presence might simply reflect differences in bone size and strength requirements (37). In addition, zebrafish HZ chondrocytes are converted into osteoblasts at the resorption front, become part of the diaphyseal endosteum and differentiate into osteocytes embedded in the bone shaft (24). This supports the presence of endochondral ossification in zebrafish in the form of (1): a thin layer of bone matrix at the resorption front and (2) bone matrix deposition inside the bone shaft, instead of the bone spongiosa described in mammals and larger teleosts (24, 32, 37). Finally, zebrafish endochondral bones do not form a marrow that can support hematopoiesis. This instead occurs in the kidney marrow of zebrafish (38).

### 2.3 Anatomical distribution of endochondral growth zones

Rodents and humans have homologous skeletal GZs inherited from a shared common ancestor, as exemplified by long bone GPs such as the proximal tibial GP. Though zebrafish GZs are not individually homologous to any mammalian GZ, a growing body of research has revealed striking similarities in their GZ development and physiology. This demonstrates the relevance of zebrafish for understanding basic principles of skeletal biology and underlying causes of skeletal disease, including common chondrodysplasias associated with GPs. These similarities include the molecular and cellular mechanisms underlying endochondral differentiation. The genetic advantages of the zebrafish, along with its small size and optical accessibility, has led to a growing popularity for their use in testing new disease candidates discovered in humans and elucidating their mechanisms of action.

#### 2.3.1 Bidirectional endochondral growth zone locations

Postembryonic growth of the human cranial base requires three bidirectional GZs: the spheno-ethmoidal, intersphenoid and spheno-occipital synchondroses (**Figure 3A**). Their importance in shaping the adult face is exemplified by the prominent forehead and flattened bridge of the nose associated with achondroplasia, the most common form of human dwarfism (39, 40). Reduced cell proliferation in the RZ of these GZs in achondroplasia results in reduced cranial base growth in patients, in addition to shortening of their arms and legs due to GP defects (**Figure 3B**) (41). The other anatomical location where bidirectional growth zones are found in humans are the vertebrae. Neurocentral synchondroses contribute to the growth of the vertebral body as well as the spinal canal (**Figure 3B**), and they fuse between ages 5 to 17 depending on their anterior-posterior location (42).

Zebrafish bidirectional growth zones are primarily located in the neurocranial and pharyngeal skeletons. As in mammals, the zebrafish neurocranium consists of both intramembranous and endochondral bones and numerous neurocranial synchondroses arise after the initial stages of chondrocranial ossification, yet their GZ activity has only recently been investigated (27). Growth of the zebrafish pharyngeal skeleton is supported by both uni- and bidirectional growth zones (**Figure 3C**). The pharyngeal skeleton derives from the pharyngeal arches (PA), which form by bilateral segmentation of the embryonic pharynx in vertebrates and their close relatives (16, 43, 44). Here we describe the PA-derived bidirectional GZs of the first (PA1, mandibular) and second (PA2, hyoid) arches, which develop first and produce the most skeletal growth, as these are most relevant to model human GZs in health and disease. For a more complete list of zebrafish pharyngeal GZs, see (27). In the dorsal PA1 skeleton, the palatoquadrate (PQ) synchondrosis mediates growth of the quadrate (QA) ventrally and metapterygoid (MP) dorsally (**Figure 3C**). In the dorsal PA2 skeleton, the hyosymplectic (HS) synchondrosis mediates growth of the symplectic (SY) ventrally and hyomandibular (HM) dorsally. In the ventral PA2 skeleton, the ventral ceratohyal (CH) synchondrosis mediates growth of the hypohyal (HH) bones ventrally and the CH dorsally, while the dorsal CH synchondrosis mediates growth of the CH (anterior CH) ventrally and epihyal (EH; posterior CH) dorsally (**Figure 3C**). In the PA3-6 (branchial arches 1-4) skeleton, basibranchial (BB) elongation is mediated by 2 bidirectional GZs (**Figure 3C**) (27).

The zebrafish PQ and CH synchondroses have been used to study developmental mechanisms that regulate GZ development (24, 26, 45, 46). These studies have shown that, like mammalian GPs, these GZs contain similar zones of cartilage maturation (RZ, PZ, HZ), though with some interesting differences in the timing of proliferation and hypertrophy. In addition, they share similar patterns of gene expression known to control GZ formation and size, as discussed below.

#### 2.3.2 Unidirectional endochondral growth zone locations

In humans, unidirectional GZs are primarily found in the limbs and ribs (**Figures 3B, D**). Growth of ribs is mediated by GZs located within costochondral joints, which are synchondroses linking ribs to the costal cartilages of vertebrae (**Figure 3B**) (47, 48). In the limbs, epiphyseal GPs mediate elongation of the stylopod (humerus, femur) and zeugopod (radius/ulna, tibia/fibula) at the end of each long bone. In contrast, each bone of the autopod grows at a single GP (phalanges/metacarpals/metatarsals; **Figure 3D**).

Zebrafish unidirectional GZs are primarily found in the pharyngeal skeleton and fin endoskeleton (**Figures 3C, E, F**). In the PA3-6 (branchial arches 1-4) skeleton, the ceratobranchial (CB) and epibranchial (EB) bones of each arch possess a unidirectional GZ at each extremity (**Figure 3C**) (27). In the 2 sets of paired fins (pectoral and pelvic) the endoskeleton is reduced compared to that of human limbs, and its proximo-distal pattern is simplified. The endoskeleton of pectoral fins consists of 4 proximal radials and 6 to 8 distal radials (**Figure 3E**), while the pelvic fins contain 3 radials (22). The caudal fin endoskeleton consists of the pleurostyle of the caudal-most vertebrae, five hypurals, the parhypural, and the haemal spines of preural vertebrae 2 and 3 (**Figure 3F**) (49). Just as in mammalian limbs, all fin GZs are unidirectional. These are positioned at the distal ends of (1) proximal radials in the pectoral, dorsal and anal fins (2), radials in the pelvic fins, and (3) hypurals, parhypural and haemal spines in the caudal fin (**Figures 3E, F**) (50). Interestingly, mutations in conserved regulators of appendage development can lead to supernumerary bones in zebrafish consistent with radials and long bones having evolved from homologous structures in the common ancestor (23).

## 3 Development and cellular architecture of endochondral growth zones in teleost fish and humans

### 3.1 Developmental similarities and differences in endochondral growth zones between species

The stereotypical steps of mammalian endochondral long bone formation consist of: 1) mesenchymal condensation, 2) differentiation into cartilage, 3) formation of a perichondral bone collar at the diaphysis and concomitant hypertrophy of chondrocytes coupled with cartilage matrix mineralization, 4) blood vessel invasion, hypertrophic chondrocyte death and resorption of mineralized matrix by chondroclasts, all at the diaphysis 5) replacement of cartilage by endochondral bone and marrow, 6) appearance of distinct RZ, PZ, and HZ zones at each epiphysis, and lastly 7) epiphyseal formation of secondary ossification centers (2, 12). These features of GZs are largely conserved between teleost fish and tetrapods, at both the cellular and molecular levels, despite the later invasion of blood vessels in teleosts, lack of hematopoietic bone marrow or secondary ossifications. Notably, endochondral bone formation in smaller teleosts, such as zebrafish takes the form of (1) a thin layer of bone matrix at the resorption front and (2) bone matrix deposition on the inner surface of the bone shaft (24, 29, 30, 32, 36, 51).

#### 3.1.1 From condensation to cartilage template

**Figure 4:**
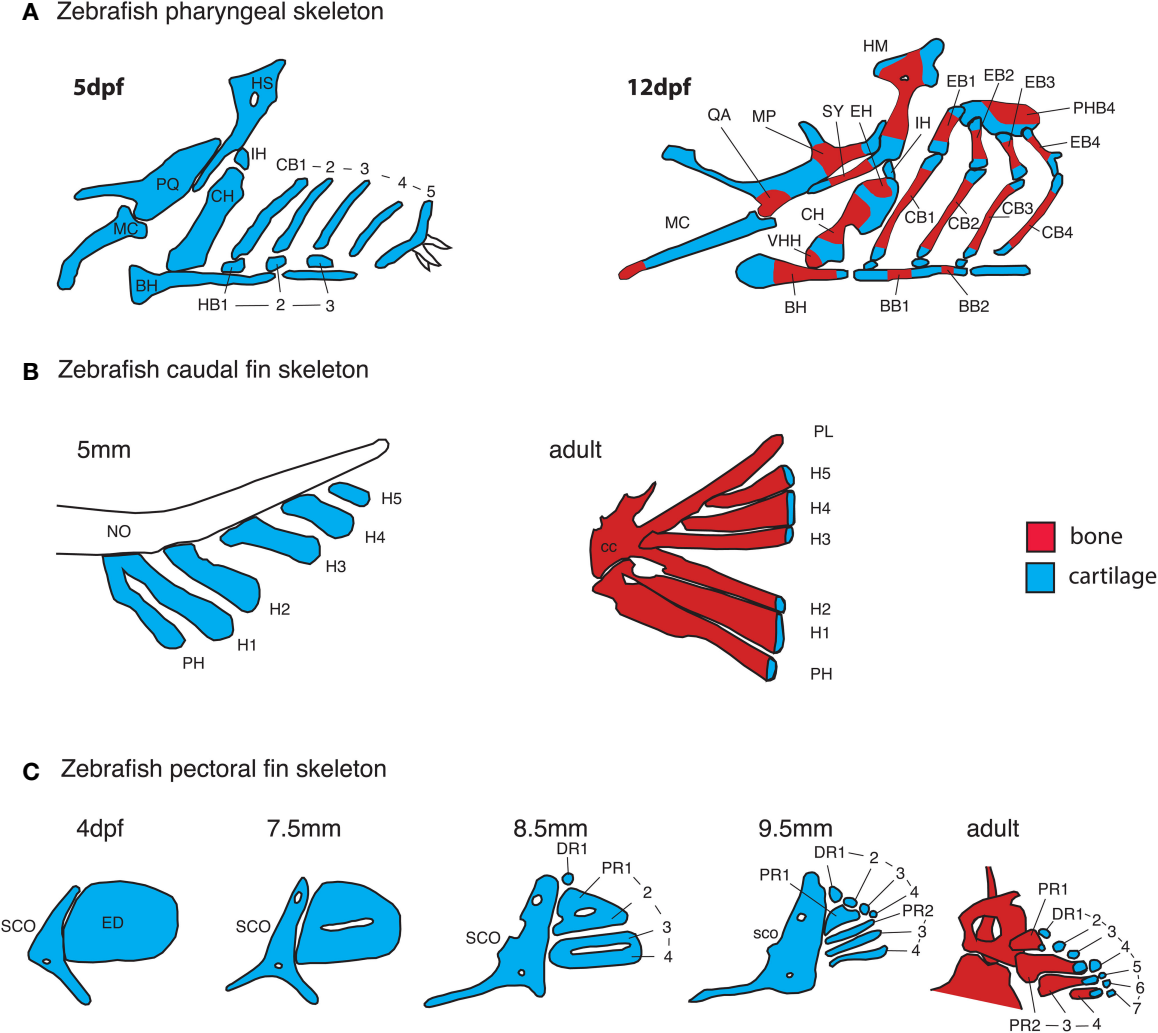
Early anatomy of zebrafish endochondral growth zones. **(A)** Endochondral growth zones start to appear in the zebrafish skeleton around 12days post-fertilization (dpf). One or more ossification centers appear on each bone. Unossified cartilage regions at bone ends become unidirectional growth zones, while those flanked by ossifying cartilage become bidirectional growth zones. In the 12dpf pharyngeal skeleton, the QA and MP bones ossify over the PQ cartilage, the HM and SY bones ossify over the HS cartilage, the HH, CH and EH bones ossify over the CH cartilage, BB1 and 2 ossify over the BB cartilage. Single ossifications appear on other pharyngeal bones. **(B)** In the caudal fin endoskeleton, single ossifications appear on each cartilage element, resulting in a single distal endochondral growth zone per element. **(C)** In the pectoral fin endoskeleton, the endoskeletal disc is progressively carved into four proximal- and seven distal radials. Ossification of each proximal radial leaves a single endochondral growth zone at the distal end. Distal radials do not ossify. BB, basibranchial; BH, basihyal; CB, ceratobranchial; CC, compound centrum; DR, distal radial; ED, endoskeletal disc; H, hypural; HB, hypobranchial; IH, interhyal; MC, Meckel’s cartilage; NO, notochord; PH, parhypural; PHB, pharyngobranchials; PR, proximal radial; SCO, scapulocoracoid, VHH, ventral hypohyal.

In tetrapods, the shape of the mesenchymal condensation determines the shape of the cartilage model (52). In contrast, cartilage elements differentiate within larger condensations in both the head and fins of teleosts (22, 53–55). Zebrafish embryonic and larval cartilage shapes generally prefigure the shape of adult skeletal elements (**Figures 4A, B**). One exception is the endoskeleton that supports the pectoral fins, in which a transient endoskeletal disc of cartilage supports the functional larval fin, but localized cartilage decomposition within the disc defines four proximal radials that prefigure the adult fin endoskeleton (**Figures 3E**, **4C**) (56).

The shapes of pharyngeal cartilage elements in teleost embryos are regulated by complex morphogenetic cell behaviors such as localized cell-cell intercalations that take place hours before cartilage matrix deposition (56–59). Linear stacking of chondrocytes driven by such intercalations underlies the directionality of the GP or GZ as well as cartilage and bone elongation. Cartilage elements of mutants with cell-cell intercalation defects are shorter and wider than in wild-type individuals (26, 60). A growing body of research supports conserved control of cell-cell intercalation during cartilage morphogenesis in the RZs of vertebrate GPs (including mammals) by planar cell polarity (PCP) pathways (26, 58, 60–66).

Though initially studied in the context of epithelia, it has become clear that noncanonical Wnt/Wnt-PCP and Fat-Dchs/Fat-PCP signaling play important roles in regulating cell and tissue polarity in diverse cell and tissue types, including mesenchyme and cartilage. Several human syndromes that affect skeletal morphology are caused by mutations in Wnt-PCP and Fat-PCP signaling genes (67–77). Studies in zebrafish have successfully modeled craniofacial defects associated with loss-of-function of *gpc4, frizzled, wnt5b*, *fat3a* and *dchs2* in cartilage morphogenesis, and demonstrated requirements for these factors in mediating the polarized cell-cell intercalation of chondrocytes in the craniofacial skeleton (26, 58, 63, 64, 66).

#### 3.1.2 Maturation of endochondral bones

The first signs of GZ development in the craniofacial skeleton in zebrafish are the simultaneous appearance of a perichondral bone collar and flattening of presumptive PZ chondrocytes during early metamorphosis (Standard Length = 6-7 mm) (26, 27). Unlike mammalian GZs, hypertrophic chondrocytes in zebrafish only enlarge slightly and transiently during zebrafish GZ development. Blood vessel invasion of the cartilage template coincides with the onset of HZ cell apoptosis, but unlike in mammals, it starts well after the onset of bone collar formation and GZ-mediated bone elongation (24, 27). It was long thought that osteocytes replacing HZ chondrocytes in GPs were introduced in the bone diaphysis by invading vasculature (2), but histological studies in chick and more recent lineage analyses using transgenic mice have shown a contribution to trabecular bone by HZ chondrocytes themselves (78–81). Similarly, recent clonal analysis using zebrafish transgenics has shown that HZ chondrocytes may undergo several fates at the resorption front: apoptosis, or transition into osteoblast or adipocyte fates (24). Unlike mammals, but similar to amphibians, reptiles and most bird species, secondary ossification centers do not develop in GZs of endochondral bones in zebrafish or other teleosts (1, 27, 29, 30).

#### 3.1.3 Patterning of endochondral growth zones

**Figure 5:**
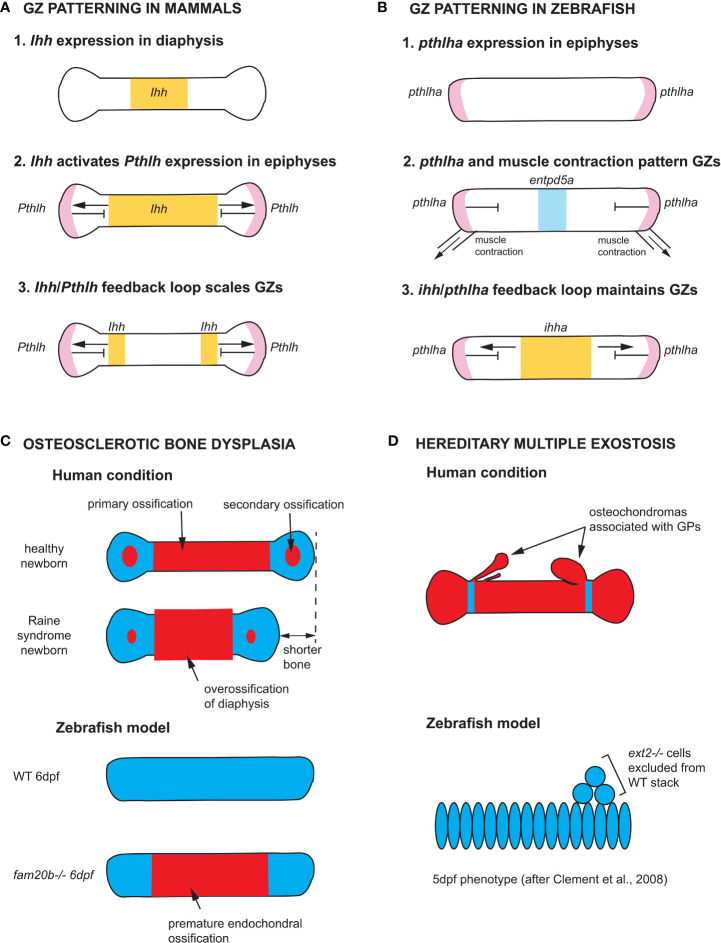
Zebrafish models of endochondral growth zone development and disease **(A)** Model for the patterning of growth zones (GZs) based on genetic studies of mouse long bones (82–84). *Indian hedgehog (Ihh)* is first expressed in the nascent diaphysis of the cartilage model. Its expression domain expands towards the epiphyses and activates *Parathyroid hormone-like hormone* (*Pthlh*) expression in periarticular cartilage. Pthlh represses Ihh at a distance, which sets the distance between the hypertrophic zone (HZ) and resting zone (RZ). **(B)** Model for the patterning of CH GZs in zebrafish based on (46). *pthlha* is expressed at the epiphyses of the differentiating CH. The HZ is then patterned by *pthlha* and muscle contractions before the onset of *ihha* expression. According to this model, *ihha* plays a role in the maintenance of GZs, not their patterning. **(C)** Zebrafish *fam20b* mutants recapitulates the skeletal phenotype of Raine syndrome, a particular form of osteosclerotic bone dysplasia (45). Short overossified long bones are observed in Raine syndrome newborns. Premature ossification of the CH diaphysis is observed in zebrafish *fam20b* mutants. **(D)** Zebrafish chimaeras recapitulate the formation of cartilage nodules observed in the human condition hereditary multiple exostosis, which results from a mutation in the *EXT2* gene. *ext2-/-* chondrocytes are excluded from WT cartilage stacks in zebrafish chimaeras, leading to the hypothesis that osteochondromas observed in *EXT2+/-* patients result from loss-of-heterozygosity (85). *Ihh* and *ihha* expression domains in yellow, *Pthlh* and *pthlha* expression domains in pink, *ectonucleoside triphosphate diphosphohydrolase 5a* (*entpd5a*) expression domain in light blue, cartilage in blue, and bone in red. CH, ceratohyal; GPs, growth plates; WT, wild type; dpf, days post-fertilization.

Our understanding of GZ patterning mechanisms is largely based on studies of mouse limb GPs. Two signaling pathways activated by Indian Hedgehog (Ihh) and Parathyroid Hormone-like Hormone (Pthlh), respectively, interact at a distance to pattern long bone GPs (**Figure 5A**). *Ihh* is first expressed throughout the diaphysis of long bone cartilage templates before becoming restricted to chondrocytes in the pre-hypertrophic zone (PHZ) (82, 86). Ihh activates *Pthlh* expression at a distance in periarticular chondrocytes, and Pthlh in turn represses *Ihh* expression. Mosaic analyses of *Ihh*, *Pthlh*, and *Pth1r* mutants have shown that this negative feedback loop effectively patterns the distance between RZ and HZ (83, 84). *Ihh* expression levels are also regulated by BMP and FGF signaling: *Smad1/5* promotes *Ihh* expression, while *Smad2/3* and *Fgfr3* repress its expression (87–90). In addition to their role in scaling the GP, Ihh promotes bone collar formation by inducing the differentiation of osteoblasts in the perichondrium (91, 92), while Pthlh promotes the proliferation of PZ chondrocytes and delays cell-cycle exit and the onset of chondrocyte enlargement, both in mice and zebrafish (82, 92). In contrast, little is known about the molecular pathways regulating HZ chondrocyte enlargement. Three phases of enlargement have been identified in mice, which include an initial three-fold volume increase through hypertrophy, that is, cell enlargement with a corresponding increase in organelle dry mass, followed by a four-fold increase through vacuole swelling by disproportionate intake of fluid, and a final two-fold increase through hypertrophy again. Interestingly, the duration of the last phase (hypertrophy) varies the most between rapidly and slowly expanding growth plates, and regulation of this phase requires *Insulin-like growth factor 1* (*Igf1*) (93).

The Ihh-Pthlh feedback loop appears to be conserved in mammalian cranial base synchondroses, although *Pthlh* is expressed throughout the RZ and PZ (94, 95). A few studies in zebrafish have shown the conservation of GZ patterning mechanisms between teleost fish and mammals (45, 96), and an earlier onset of *Pthlha* expression than previously described, namely at the onset of chondrogenesis and before the onset of *ihha* expression (**Figure 5B**) (46). Novel findings in zebrafish have also shown the potential of this model for expanding our understanding of GZ patterning, as they suggest that the Ihh-Pthlh feedback loop maintains but does not establish the GZ pattern, at least in some pharyngeal endochondral bones (46). Instead, the zebrafish *Pthlh* ortholog, *pthlha*, and mechanical force from muscle contraction initiate the HZ and the location of subsequent *ihha* expression, thereby establishing the negative feedback-loop that maintains GZs (**Figure 5B**) (46).

### 3.2 Cellular basis of similarities and differences in endochondral growth zones between species

#### 3.2.1 Bone elongation and differential growth

The rate of bone elongation changes throughout the life of a GZ, and differs between GZs of an individual, as well as homologous GZs of different species. Such growth rate variation is referred to as differential growth (1). In rats, three cellular mechanisms mediate endochondral bone elongation: cell proliferation, cell enlargement, and ECM production. Cell proliferation takes place in the PZ and enlargement in the HZ, while ECM production takes place in both zones. These three cellular mechanisms contribute unequally to bone elongation in mammalian GPs: proliferation 7-10%, ECM production 32-49%, and cell enlargement 44-59% (25). The relatively minor contribution of proliferation to growth serves to compensate for the loss of chondrocytes at the chondro-osseous junction. Between mammalian species, as shown for bat metacarpal and jerboa metatarsal GZs, the largest driver of growth rate is the degree of cell enlargement of HZ chondrocytes (93, 97). In contrast, proliferation is the major contributor to endochondral bone elongation in zebrafish, as no significant cell enlargement or increase in ECM content are observed in active GZs (27). In other teleost fishes, the cellular basis of endochondral growth has been explored in several African cichlids: ECM production is the main driver of growth in *H. elegans*, while differences in cell proliferation and/or enlargement mediate differential growth in Lake Malawi cichlids (98, 99).

#### 3.2.2 Life history differences

Mammals and teleost fishes also differ dramatically in the timing of growth over their lifespans. Human limb GPs are already active at birth and mediate axial elongation until the end of puberty, when estrogens trigger GP closure and growth arrest through complete replacement of epiphyseal cartilage by bone (100). In rats, GPs also become inactive at sexual maturity but they are not replaced by bone (1). Not all GPs become inactive at the same age: in humans, the three GZs of the cranial base ossify at different times: the intersphenoid GZ ossifies immediately before birth, the spheno-ethmoidal GZ ossifies at 6 years, and the spheno-occipital GZ remains active until the end of puberty (101–103). In contrast, most teleost fish grow throughout life, although the rate of growth slows with age, as described by the individual growth model of von Bertalanffy (104). Accordingly, zebrafish growth is indeterminate (105, 106), yet its pharyngeal GZs become inactive in adults and do not ossify, similar to rats. Further adult growth is mediated by intramembranous ossification (27).

### 3.3 Modeling human endochondral growth zone disorders in zebrafish

Despite the many similarities in development and physiology of their GZs, there have been relatively few studies modeling human GZ disorders in zebrafish. Recent reviews largely focus on the many models for other types of bone diseases such as osteogenesis imperfecta, osteoporosis, osteopetrosis and osteoarthritis, that alter ossification and osteoblasts (4–10). Notable exceptions include mutations in genes encoding proteoglycan core proteins or enzymes involved in their biosynthesis or assembly, as models for such cartilage diseases as Keipert syndrome (Glypican 4, GPC4; discussed in section 1a), osteosclerotic bone dysplasia (FAM20C), and hereditary multiple exostoses (Exostosin 2, EXT2).

Zebrafish provided some of the first insights into requirements for proteoglycans in craniofacial development (45, 96). A variety of Human conditions result from mutations in the proteoglycan biosynthesis pathway that builds chondroitin-sulfate- (CSPGs) and heparin-sulfate- proteoglycans (HSPGs) from UDP-glucose. Zebrafish mutants in seven of the nine enzymes of the O-linked-glycosylation pathway required for HSPG production have been described, several of which recapitulate endochondral skeletal defects of human patients (107).

A surprising discovery associated with the cartilage phenotypes of zebrafish mutants in *UDP-xylose synthase* (*uxs1*), *xylotransferase 1* (*xylt1*) and *glycosaminoglycan xylosyl kinase* (*fam20b*) is the premature maturation of hypertrophic chondrocytes and bone collar ossification (**Figure 5C**). This suggested a role for proteoglycans in regulating the timing of cartilage and bone differentiation, perhaps through the modulation of ligand-based cell-cell signaling (45, 96). Further, premature ossification in *fam20b* mutants provided a new etiology for Raine syndrome, a human disease resulting from mutations in *FAM20C*. Also known as osteosclerotic bone dysplasia, Raine syndrome patients have craniofacial defects such as low nasal bridge and midfacial hypoplasia indicative of defects in growth at synchondroses, as well as short and overossified long bones in newborns (**Figure 5C**). The zebrafish *fam20b* mutant phenotype suggests that Raine syndrome craniofacial and limb skeletal defects result from premature maturation of the skeleton (45).

Further down the HSPG biosynthetic pathway, *exostosin* (*ext*) *-1 and-2* code for glycosyltransferases involved in the polymerization of heparan sulfate chains. Mutations in *EXT1* or *EXT2* result in hereditary multiple exostoses (HME) in humans, a disease that causes the formation of benign bone tumors (osteochondromas) that are associated with GPs (**Figure 5D**). Most HME patients are heterozygous for mutations in either *EXT1* or *EXT2* (108–110). A study of zebrafish *ext2* mutants (*dackel*) supports a model where osteochondromas arise from local loss of heterozygosity (LOH): zebrafish *ext2^-/-^* embryos do not develop osteochondromas but their skeleton is generally misshapen, demonstrating a requirement for *ext2* in cartilage morphogenesis/stacking (85). Instead, *ext2^-/-^* cells form osteochondroma-like nodules when transplanted in wild type (WT) individuals: homozygous mutant cells are excluded from WT stacks, providing support to the LOH model for the etiology of HME (**Figure 5D**) (85).

## 4 Conclusions and future directions

In this review, we have highlighted the many similarities and differences between zebrafish and human skeletal anatomy, their endochondral GZs and recent studies of developmental and physiological mechanisms that control endochondral bone growth. Despite the apparent anatomical differences between human and teleost fish skeletons, the overwhelming conservation of different cell types and molecular mechanisms underlying skeletal development makes the zebrafish a powerful model for further studies of the causes and potential therapies for human skeletal diseases. This power lies in (1): the unique and well-known properties that have already made zebrafish a popular model system, which include ease of care, their small size, large number of offspring, suitability for large forward genetic screens and embryo transparency to name a few and (2) an ever-expanding toolkit to reach a diversity of research goals. CRISPR-Cas9-mediated mutagenesis is relatively easy in zebrafish and protocols have been developed for the rapid production of loss-of-function phenotypes in CRISPR-injected individuals (111, 112). Numerous transgenic lines labeling various skeletal cell lineages and their precursors have been used to image cartilage and bone morphogenesis *in vivo*, and also conduct lineage tracing in endochondral bones (24, 58, 113, 114). Transgenic zebrafish can also be utilized for cell-type and stage-specific ablation using the nitroreductase system (115), as well as in mosaic transgenic conditions to test the cell-autonomous and non-cell autonomous properties of particular genes and their mutant alleles (46, 116, 117). Lastly, recent improvements in single-cell RNAseq and ATACseq methodologies have allowed gene expression profiling of entire cell lineages and even whole organs or organisms at single cell resolution, made possible by the small size of zebrafish embryos and larvae (118–122). Future deployment of these single-cell techniques for the study of all skeletal cell types will undoubtedly lead to new insights into endochondral and GZ development in health and disease.

## Author contributions

PL, DD, DH and TS all contributed to the conception and content covered in the manuscript. PL wrote the first draft of the manuscript. TS wrote sections of the manuscript. All authors contributed to manuscript revision, read, and approved the submitted version.
